# DMPat-based SOXFE: investigations of the violence detection using EEG signals

**DOI:** 10.1007/s11571-025-10266-6

**Published:** 2025-06-05

**Authors:** Kubra Yildirim, Tugce Keles, Sengul Dogan, Turker Tuncer, Irem Tasci, Abdul Hafeez-Baig, Prabal Datta Barua, U. R. Acharya

**Affiliations:** 1 Department of Digital Forensics Engineering, Technology Faculty, Firat University, Elazig, Turkey; 2 Department of Neurology, School of Medicine, Firat University, Elazig, Turkey; 3 School of Business, University of Southern Queensland, West Street Toowoomba, Springfield, QLD Australia; 4 School of Business (Information System), University of Southern Queensland, Springfield, Australia; 5 School of Mathematics, Physics and Computing, University of Southern Queensland, Springfield, Australia

**Keywords:** SOXFE, Distance matrix pattern, Violence detection, Cortical connectome diagram, EEG signal analysis

## Abstract

Automatic violence detection is one of the most important research areas at the intersection of machine learning and information security. Moreover, we aimed to investigate violence detection in the context of neuroscience. Therefore, we have collected a new electroencephalography (EEG) violence detection dataset and presented a self-organized explainable feature engineering (SOXFE) approach. In the first phase of this research, we collected a new EEG violence dataset. This dataset contains two classes: (i) resting, (ii) violence. To detect violence automatically, we proposed a new SOXFE approach, which contains five main phases: (1) feature extraction with the proposed distance matrix pattern (DMPat), which generates three feature vectors, (2) feature selection with iterative neighborhood component analysis (INCA), and three selected feature vectors were created, (3) explainable results generation using Directed Lobish (DLob) and statistical analysis of the generated DLob string, (4) classification deploying t algorithm-based k-nearest neighbors (tkNN), and (5) information fusion employing mode operator and selecting the best outcome via greedy algorithm. By deploying the proposed model, classification and explainable results were generated. To obtain the classification results, tenfold cross-validation (CV), leave-one-record-out (LORO) CV were utilized, and the presented model attained 100% classification accuracy with tenfold CV and reached 98.49% classification accuracy with LORO CV. Moreover, we demonstrated the cortical connectome map related to violence. These results and findings clearly indicated that the proposed model is a good violence detection model. Moreover, this model contributes to feature engineering, neuroscience and social security.

## Introduction

Detection of violence with electroencephalography (EEG) signals is an approach that investigates the neurophysiological basis of violence and aggressive tendencies in individuals by analyzing electrical activity in the cerebral cortex (Bars et al. 2001; Patrick 2008). EEG analysis examines the frequencies of brain waves and activity changes in brain regions (Calzada-Reyes et al. 2013; Weon et al. 2021). In this way, information about violent tendencies can be obtained (Aliyari et al. 2020).

The frontal cortex and brain sub-regions related to emotional control play an important role in violent tendencies (Blair 2017; Siever 2008). The frontal lobe, especially the orbital frontal cortex (OFC) and dorsolateral prefrontal cortex (DLPFC) are areas that regulate the individual's impulse control, decision-making and social behaviors (Nejati et al. 2018). While the DLPFC plays a role in cognitive control and attention processes, the OFC is involved in regulating emotional reactions and evaluating social behaviors (Lin and Feng 2024). Neurological activity abnormalities are observed in these regions in individuals prone to violence. Aggressive and violent tendencies can be detected by analyzing the electrical activities of these regions with EEG (Golden et al. 1996; Hoptman 2003).

Beta and gamma frequencies are associated with high cognitive workload and intense emotional processes. Increases in this frequency band can be seen in severe emotional situations. For example, beta waves (13–30 Hz) may increase in stress, anxiety and arousal, while gamma waves (30 Hz and above) can be associated with emotional arousal and aggressive behavior (Chikhi et al. 2022). The increase observed in these frequencies in EEG signals may indicate that the individual may be prone to violence at the neurophysiological level (Patrick 2008). In addition, left and right frontal lobe asymmetry is an important factor in the detection of violence with EEG (Rohlfs and Ramírez 2006). Frontal alpha asymmetry is a condition frequently observed in individuals with a tendency to violence (Lake et al. 2014). Low activity in the left frontal region, in particular, can be associated with negative emotional states and aggression (López-Castro et al. 2023). This situation may cause a decrease in the individual's emotional control and social adaptation capacity (Stead et al. 2023; Zsigo et al. 2024). Such EEG analyses can be used in clinical assessment and diagnosis processes in psychiatry (Hassan et al. 2023; Squires et al. 2023), forensic sciences (Fidas and Lyras 2023; O'Brien et al. 2024), and neuropsychology (Carrarini et al. 2024). In addition, EEG-based machine learning models can help predict and classify an individual's violent tendencies with biomarkers extracted from EEG data (Othmani et al. 2023). For example, explainable AI (XAI) methods such as Directed Lobish can contribute to these detection processes by making EEG signals more understandable (Tuncer et al. 2024b).

### Literature review

The literature contains many machine learning methods (Gorur 2023; Gorur and Eraslan 2022; Gorur et al. 2023; Ozturk et al. 2025). Below are some of the research on violence detection (VD). Huszár et al. (2023) developed a VD model in video surveillance. They used public datasets, including Crowd Violence and Hockey Fights. They achieved approximately accuracies of 80% across these datasets. Yildiz et al. (2023) proposed an audio-based VD approach using the TreePat23 feature extraction (FEX) method on a dataset of 1444 audio observations collected from YouTube. They achieved classification accuracies of 89.68% with kNN and 89.75% with SVM for leave-onerecord-out cross-validation. Rendón-Segador et al. (2023) developed CrimeNet for video VD. Tested on datasets like UCF Crime and XD Violence, they attained an accuracy of 99.98%. Kaur and Singh (2024) presented an analysis of vision-based VD in videos using hybrid methods. They evaluated various approachs on datasets like Hockey Fight, Movies, and Crowd Violence. The numerical results indicated varying levels of success across methods. Tang et al. (2024) proposed a VD system for animations using a modified Faster R-CNN approach. They utilized a dataset of 4,044 violent images. They achieved an average precision of 79.78%. Ehsan et al. (2023) developed an unsupervised VD framework using a double-stream AutoEncoder. They used the Hockey and Movies datasets for evaluation. They calculated 84% and 98% accuracy. Durães et al. (2023) proposed an audio-based VD system in cars using a custom dataset. They tested various approachs and found that EfficientNetB1 achieved the highest accuracy at 95.06%. Bakhshi et al. (2023) presented a VD system using lightweight deep neural networks on audio signals. They reported that their best-performing lightweight approach achieved an accuracy of 96.59% on the Real-Life Violence Situations dataset. Garcia-Cobo and SanMiguel (2023) suggested a VD approach using human skeleton extraction and change detection. They tested it on the RWF-2000 dataset, achieving 90.25% accuracy. Park et al. (2024) proposed a Conv3D-based VD approach with optical flow, RGB data to capture spatiotemporal features in videos. They utilized the Hockey, UBI-Fight, Movie-Fights, Crowd datasets for evaluation and attained high area under the curve (AUC) scores of 95.4, 98.1, 94.5, and 100.0 on these datasets. Abbass and Kang (2023) developed an enhancement for VD using the UBI-Fights dataset with deep learning architectures integrated with convolutional block attention modules. They addressed class imbalance using categorical focal loss and achieved an AUC of 94.93%. Magdy et al. (2023) presented a 4D CNN model for VD in surveillance videos using four benchmark datasets. The approach achieved 94.67% accuracy on RWF2000, 97.29% on Crowd Violence, and 100% on both Movie Fight, Hockey Fight datasets. Rahman et al. (2023) suggested a method to test the robustness of VD in videos using the Hockey Fight, Movie datasets. They attained high attack success rates of 86.84% and 87.50%. Aldehim et al. (2023) suggested a VD method using the tuna swarm optimization with deep learning. Tested on the Hockey Fight, Movies datasets, their approach achieved high accuracy rates of 98.72% and 98.44%.

### Research gaps

- Most researchers have used well-known deep learning (DL) or feature engineering (FE) approaches for EEG signal classification (Khalfallah et al. 2025; Moctezuma et al. 2025). This situation causes stagnation in the presentation of new generation approaches for EEG signal classification.
- DL models have exponential time complexity, while FE approaches have linear time complexity. While DL models attained high classification performance, FE models have reached relatively lower classification performance (Chen et al. 2024; Shirodkar et al. 2025; Talukder et al. 2024). This tradeoff should be addressed.
- There are limited explainable FE models, and most FE models have focused on attaining high classification performance (Wang et al. 2025).

### Motivation and our approach

Our primary motivation is to fill the gaps in the existing literature. Therefore, we published an EEG VD dataset.

To classify these EEG signals, we suggested a new FE approach, inspired by transformers and k-nearest neighbors. The presented FE approach has five phases, which are:

- DMPat-based feature extraction (FEX),
- Feature selection (FS),
- DLob-based XAI results generation,
- tkNN (Tuncer et al. 2024b)-based classification,
- Information fusion.

We suggested a new FEX function named DMPat in the FEX phase. The proposed DMPat computes the distances between channels and identifies the channels with the minimum and maximum distances. By using these channels, decimal values were created, and the histograms of the two generated feature map signals were extracted and named feature vectors. Moreover, a third feature vector was created by merging the two generated feature vectors, using the minimum and maximum distances.

INCA (Tuncer et al. 2020) was applied to these three feature vectors, and three chosen feature vectors were created in the FS phase. In the DLob phase, the indices of the chosen features were employed to create DLob strings, and cortical connectome diagrams were computed using these DLob strings. tkNN, an ensemble classifier, was used in the classification phase. Mode-based majority voting was applied, and the voted outcome was generated. By deploying a greedy algorithm, the best of the four generated outcomes (3 classifier-based + 1 voted) was selected. Using this strategy, we presented a new generation DMPat-based SOXFE approach.

The presented DMPat-based SOXFE approach has linear time complexity and achieved high classification performance. The recommended DMPat-based SOXFE approach attained 100% and 98.49% classification accuracies with tenfold and LORO CVs, respectively. These results clearly demonstrate the robustness of the DMPat-based SOXFE approach. Our approach is a self-organized approach because we used a greedy algorithm in the classification (tkNN) and information fusion phases. In this respect, tkNN selects the best outcomes from the computed parameter-based and voted outcomes. In the information fusion phase, the best outcome was selected, resulting in high classification performance with linear time complexity.

To obtain explainable results, we integrated the suggested approach with DLob. By deploying DLob, cortical connectomes were generated, providing insights related to neuroscience.

### Contributions and novelties

Contributions:

- The presented DMPat-based SOXFE is a highly accurate approach. This approach attained over 98% classification accuracy on the collected EEG VD dataset. In this respect, it contributes to FE and is competitive with DL models.
- By integrating DLob with the proposed FE approach, we generated cortical connectome diagrams. In this regard, this research contributes to neuroscience. Moreover, this research has potential to contribute social security in spite of VD.

Novelties:

- A new EEG VD dataset was collected.
- We proposed a new FEX function, named DMPat.
- By deploying the proposed DMPat as a feature extractor, a new SOXFE approach was presented.

## Dataset

In this research, a novel EEG VD dataset was utilized, and this VD dataset has two classes: (i) resting/control and (ii) violence (Bektas et al. 2024). The EEG signals was gathered from 14 participants (11 males and 3 females). During EEG collection, the meditation and violence videos were demonstrated to the subjects. To collect the EEG signals, a brain cap with 14 channels was used, and the sampling frequency of this brain cap is 128 Hz. This brain cap is Epoch X, and the channels used are: (1) AF3, (2) F7, (3) F3, (4) FC5, (5) T7, (6) P7, (7) O1, (8) O2, (9) P8, (10) T8, (11) FC6, (12) F4, (13) F8, and (14) AF4. After collecting the EEG signals, they were seperated into segments, each 15 s long (1920 = 128 × 15).

During the dataset collection phase, participants watched *two* videos. The goal was to create two emotional states: violence and rest. For the violence condition, short film clips showing conflict and aggression and violence were shown. These clips were taken from past user comments and are known to create strong emotional responses and researchers used publicly available violence videos. For the rest/control condition, meditation and nature videos were used. These videos showed calming scenes and soft music to help the participants feel relaxed.

Each session included several short clips, ranging in length of the clips was between 1 and 5 min. This was enough to record clear EEG signals. Before starting, each participant signed a consent form. This study was confirmed by the ethics board of Firat University.

The layout of the acquired EEG signals is shown in Table 1.

**Table 1:** Details of the collected dataset

No	Class	Number of records	Number of segments
1	Violence	47	286
2	Rest/Control	38	442
Total		85	728

In Table 1, "records" refers to full EEG recording sessions taken while participants watched videos. One participant could have more than one record if they joined more than one session. So, the number of records does not always match the number of participants.

Each EEG record was split into smaller parts/segments. The length of the part/segment is 15 s. Since the sampling rate is 128 Hz, each segment contains 1920 data points. The "number of segments" column shows how many 15-s pieces/segments/parts were taken from all the EEG records in each class.

For example, the violence class includes 47 records. These were divided into 286 segments. The control class has 38 records, which were divided into 442 segments.

This dataset was published on Kaggle, and can be downloaded it in.mat format using the following URL: https://www.kaggle.com/datasets/turkertuncer/turkish-violence-detection-eeg-dataset.

## The presented SOXFE

The essential objective of this SOXFE approach is to obtain high classification accuracy with explainable results. This approach has five main phases, which are:

- DMPat-based FEX,
- Feature selection based on INCA,
- Explainable results employing DLob symbolic language,
- tkNN-based classification,
- Information fusion.

**Fig. 1:**
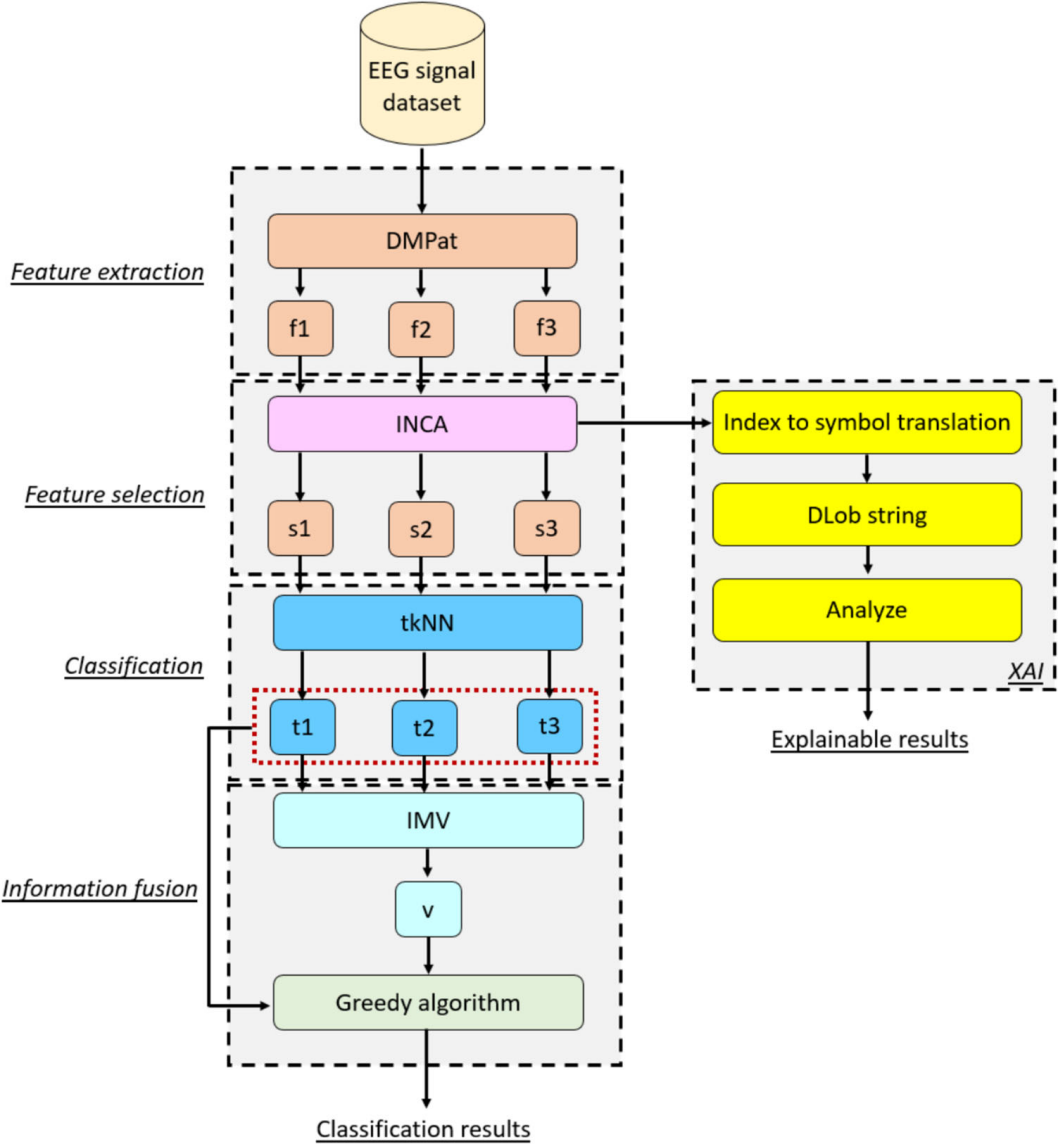
Visuıal overview of the proposed DMPat-based SOXFE approach. Herein, f: feature vector, s: chosen feature vector t: tkNN-based outcome and v: voted outcome

To clarify the presented approach, a visual depiction of this approach is showcased in Fig. 1.

A detailed explanation of the recommended DMPat-based SOXFE approach is provided below.

**Step 1:** Apply DMPat to the proposed approach to extract features.

1 $$\left[{f}_{1},{f}_{2},{f}_{3}\right]=DMPat(EEG)$$

where $DMPat(.)$: DMPat FEX function and $f$: feature vector.

**Fig. 2:**
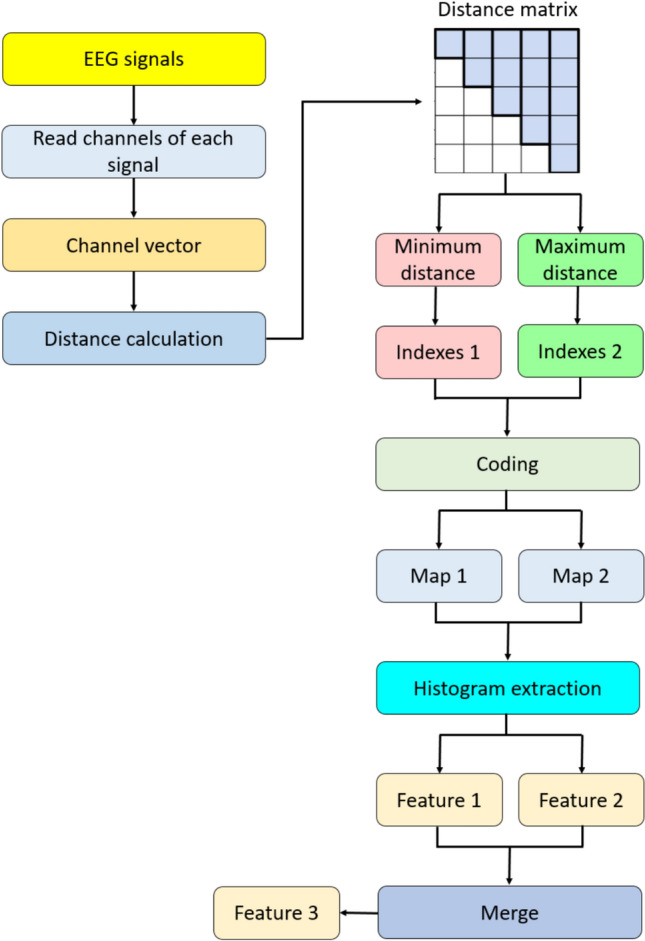
Graphical explanation of the proposed DMPat FEX function

In this step, we derived three feature vectors, and the lengths of which are 196, 196, and 392, as the third feature vector is the concatenated version of the first two feature vectors. To better explain this step, the visual depiction of this feature vector is demonstrated in Fig. 2.

The steps of the presented DMPat are also given below for clarification.

**Step 1.1:** Create channel vector by reading channels of each point.

**Step 1.2:** Compute the distance vector for each channel value and create a distance vector.

2 $$DM\left(i,j\right)=\sqrt{C{h}_{i}^{2}+C{h}_{j}^{2}}, i,j\in \left\{\mathrm{1,2},\dots ,NC\right\}$$

Herein, $DM$: distance matrix, $NC$: number of channels and $Ch$: channel value.

**Step 1.3:** Select the points with minimum and maximum distances.

3 $$\left[{a}_{1},{a}_{2}\right]=argmin(DM)$$

4 $$\left[{b}_{1},{b}_{2}\right]=argmax(DM)$$

Herein, $a,b$: channels indices. By deploying these indices, feature map signals have been computed.

**Step 1.4:** Compute the feature map values deploying the extracted channel indices.

5 $$va{l}_{1}={(a}_{1}-1)\times NC+({a}_{2}-1)$$

6 $$va{l}_{2}={(b}_{1}-1)\times NC+({b}_{2}-1)$$

Herein, $val$: the computed decimal values.

**Step 1.5:** Iterate steps 1.1–1.4 until the desired number of EEG signals is observed and value arrays are obtained.

**Step 1.6:** Extract histograms of the value arrays and obtain the first and second feature vectors.

7 $${f}_{k}=\varepsilon \left(va{l}_{k}\right), k\in \{\mathrm{1,2}\}$$

where $f$: feature vector with a length of $N{C}^{2}$, $\varepsilon (.)$: histogram extraction function.

**Step 1.7:** Merge the first and second feature vector to generate third feature vector.

8 $${f}_{3}\left(i\right)={f}_{1}\left(i\right), i\in \{\mathrm{1,2},\dots ,N{C}^{2}\}$$

9 $${f}_{3}\left(i+N{C}^{2}\right)={f}_{2}\left(i\right)$$

These seven steps (Steps 1.1–1.7) have been defined in the presented DMPat.

In the presented DMPat, meaningful features are extracted. The use of minimum and maximum distances between EEG channels is based on the functional characteristics of brain activity. Minimum distances often indicate strong functional connectivity between adjacent regions, common during focused cognitive states or emotional processing. Although maximum distances show long-range interactions between distant brain areas. These distances show how different parts of the brain work together at a global level. By combining both local and distant relationships, the DMPat FEX method captures a broad range of inter-channel dynamics. Using this strategy, we can extract the meaningful features.

**Step 2:** Chose the most informative features by implementing the INCA feature selector, generate three selected feature vectors (Tuncer et al. 2020).

10 $${s}_{k}=INCA\left({f}_{h},y\right),h\in \{\mathrm{1,2},3\}$$

Here, $s$: selected feature vector, $INCA(.)$: INCA feature selector, ${f}_{h}$: h^th^ feature vector and $y$: actual/real labels.

The steps of the INCA feature selector appear as follows.

**Step 2.1:** Derive the qualified indexes by deploying NCA feature selector (Goldberger et al. 2004).

11 $$i{d}_{h}=NCA({f}_{h},y)$$

where $id$: the qualified indexes of the by computing NCA feature selector.

**Step 2.2:** Deploy iterative feature selection.

12 $$s{f}_{h}^{r-start+1}\left(d,j\right)={f}_{h}\left(d,i{d}_{h}\left(j\right)\right), j\in \left\{\mathrm{1,2},\dots ,r\right\},$$

L2 $$r\in \{start,start+1,\dots ,stop\}, d\in \{\mathrm{1,2},\dots ,nos\}$$

Herein, $sf$: iterative selected feature vector, $start$: start index of the loop, $stop$: stop index of the loop and $nos$: number of segments.

**Step 2.3:** Derive the misclassification value of the selected features by deploying a classifier. In this research, we have used k-nearest neighbors (kNN) with tenfold CV.

13 $$loss\left(r-start+1\right)={\mathbb{C}}\left(s{f}_{h}^{r-start+1},y\right)$$

where $loss$: the computed loss values and ${\mathbb{C}}(.)$: classifier.

**Step 2.4:** Select the best feature vector with minimum loss value. We employed a self-organized feature selector called INCA.

14 $$index=argmin(loss)$$

15 $${s}_{h}=s{f}_{h}^{start+index-1}$$

where ${s}^{h}$: the ultimate selected feature vector of the h^th^ method.

**Step 3:** Generate DLob string with the indices of the chosen features. The DLob string generation phase is demonstrated in below using mathematical equations (Tuncer et al. 2024a).

16 $$Table=\{FL, FL, FL, FL, TL, PL, OL, OR, PR, TR, FR, FR, FR, FR\}$$

17 $${nid}_{1}=\lceil\frac{sfi{d}_{h}\left(t\right)}{NC}\rceil,t\in \{\mathrm{1,2},\dots ,N\}$$

18 $${nid}_{2}=sfi{d}_{h}\left(t\right) \left(mod NC\right)+1$$

19 $$dl{s}_{h}\left(c\right)=Table\left({nid}_{1}\right), c\in \{\mathrm{1,3},\dots ,2N-1\}$$

20 $$dl{s}_{h}\left(c+1\right)=Table\left({nid}_{1}\right)$$

Herein, $Table$: look up table of the employed brain cap according to DLob, $nid$: index of the DLob symbol, $sfid:$ index of the chosen feature vector, $N$: the number of the selected features, $dls$: the generated DLob string. Moreover, the meaning of the used DLob symbols are given below.

FL: The left frontal lobe is referred to, being responsible for higher cognitive functions such as reasoning, decision-making, and problem-solving.

FR: The right frontal lobe is corresponded to, playing a key role in regulating emotions, social behavior, and understanding spatial relationships.

OL: The left occipital lobe is represented, being primarily involved in interpreting visual information from the right visual field.

OR: The right occipital lobe is stood for, processing visual stimuli from the left visual field.

PL: The left parietal lobe is signified, being responsible for integrating sensory information, spatial awareness, and aspects of language comprehension.

PR: The right parietal lobe is symbolized, being crucial for processing sensory inputs and contributing to spatial orientation and body coordination.

TL: The left temporal lobe is denoted, being essential for language comprehension, auditory processing, and memory functions.

TR: The right temporal lobe is represented, being associated with processing sounds, retrieving memories, and emotional responses.

We applied information entropy analysis, built transition tables for the cortical connectome diagram, and extracted insights from the three DLob strings.

**Step 4:** Assign classes to the chosen feature vectors via tkNN.

21 $${t}_{h}=tkNN({s}_{h},y)$$

where $t$: tkNN-based outcomes, ${s}^{h}$: the ultimate selected feature vector of the h^th^ method and $y$: actual/real labels.

The tkNN is an ensemble classifier and the mathematical definition of classifier is given below (Tuncer et al. 2024a).

22 $$dv=\{cityblock,euclidean,cosine,spearman\}$$

23 $$w=\{squaredinverse,equal,inverse\}$$

24 $$k=\{\mathrm{1,2},\dots ,10\}$$

25 $$p{out}_{e}^{h}=kNN\left({S}_{g},y,{dv}_{a},{w}_{b},{k}_{c}\right),$$

26 $$a\in \left\{\mathrm{1,2},3\right\}, b\in \left\{\mathrm{1,2},\mathrm{3,4}\right\}, c\in \left\{\mathrm{1,2},\dots ,10\right\}, e\in \left\{\mathrm{1,2},\dots ,120\right\}$$

27 $$cac(e)=\phi \left({out}_{e}^{h},y\right)$$

28 $$ix=argsort(-cac)$$

29 $${vout}_{l}^{h}=\varpi \left(p{out}_{ix\left(1\right)}^{h}, p{out}_{ix\left(1\right)}^{h},\dots ,p{out}_{ix\left(l+2\right)}^{h}\right),l\in \left\{\mathrm{1,2},\dots ,118\right\}$$

30 $$cac(120+l)=\phi \left({vo}_{l}^{h},y\right)$$

31 $$idx=argmax(cac)$$

32 $${t}_{h}=\left\{\begin{array}{c}p{out}_{idx}^{h}, idx\le 120\\ v{out}_{idx-120}^{h}, idx>120\end{array}\right.$$

Herein, $dv$: distance value, $w$: weight of the kNN, $pout$: parameter-based outcome, $cac$: classification accuracy, $pout$: parameter-based outcome, $\phi (.)$: classification accuracy computation function, $ix$: the qualified classification accuracies by descending, $vout$: voted outcome and $idx$: the index of the maximum classification accuracy.

Equations (22)–(26) define the iterative parameter-based outcomes generation, Eqs. (27)–(29) explain the IMV algorithm, and Eqs. (30)–(32) represent the greedy algorithm. In this context, the tkNN used employs iterative parameter-based outcomes generation, IMV-based voted outcomes generation, and a greedy algorithm to choose the best outcome.

**Step 5:** Generate voted outcomes by deploying mode-based majority voting and select the best outcome via a greedy algorithm (DeVore and Temlyakov 1996).

33 $$vot=\varpi ({t}_{1},{t}_{2},{t}_{3})$$

34 $$fot=GA({t}_{1},{t}_{2},{t}_{3},vot)$$

where $vot$: the final voted outcome, $fot$: the ultimate/final outcome and $GA(.)$: greedy algorithm.

**Fig. 3:**
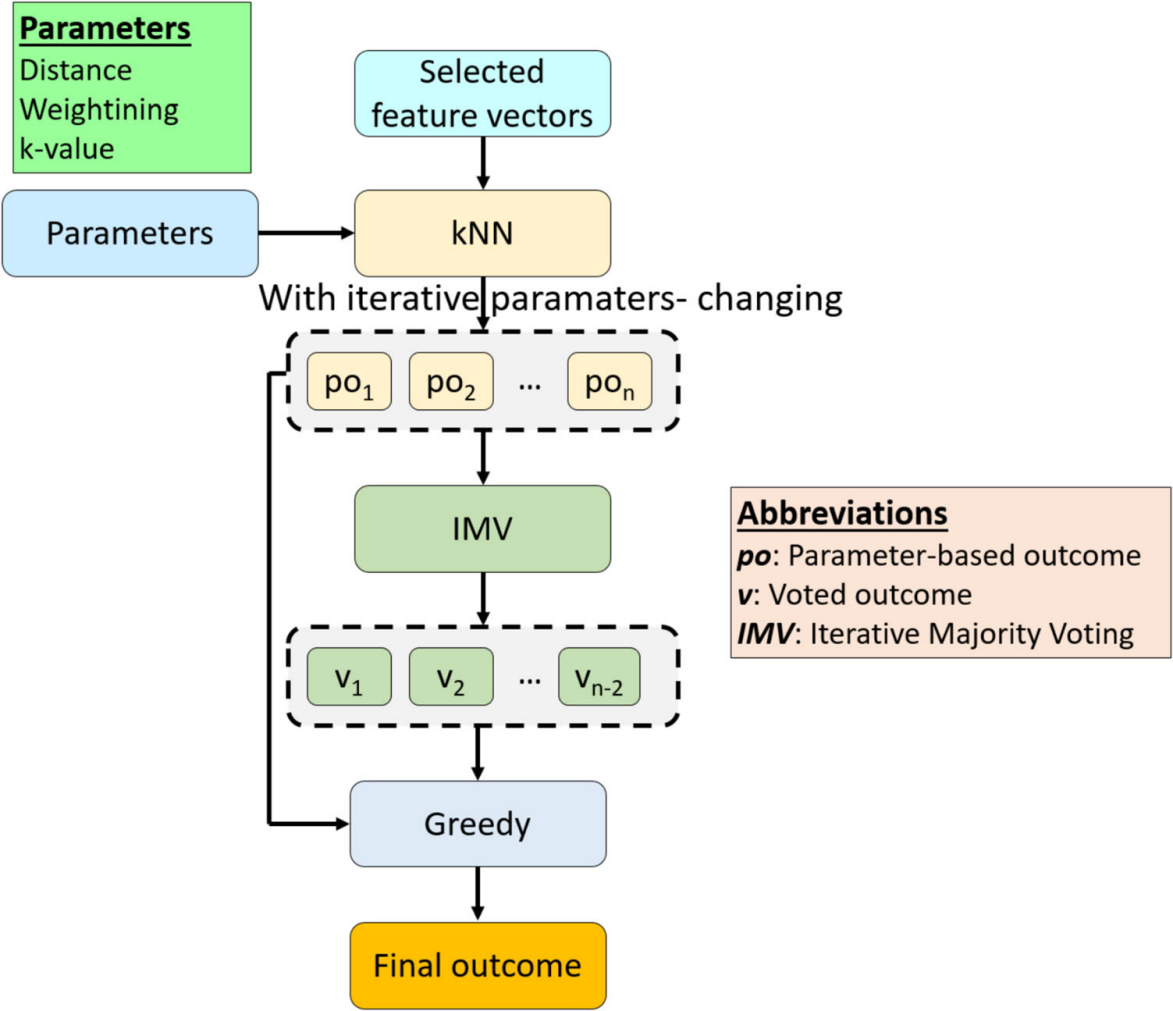
Graphical depiction of the tkNN classifier

These five steps clearly defined the presented DMPat-based EEG VD approach. The graphical outcome of the tkNN classifier is demonstrated in Fig. 3.

The Fig. 3 shows the working of tkNN classifier. We generated voted outcomes by using the IMV and parameter-based outcomes. A greedy algorithm chosen the best result by choosing the outcome with the highest classification accuracy.

## Performance evaluation

The presented DMPat-based SOXFE approach was coded via the MATLAB (version 2024a) programming environment. This SOXFE approach, has linear time complexity. Therefore, it was deployed in central processing unit (CPU) mode, without parallel programming approaches or graphical processing unit (GPU) hardware.

The initial parameters of the proposed DMPat-based SOXFE approach are given below:

FEX:

DMPat: The dataset used has 14 channels, resulting in a distance matrix of size 14 × 14. Maximum and minimum distances were used to generate the feature map. The first and second feature maps were created by extracting the histogram of these generated feature maps. The lengths of the first and second feature maps are both 196. The third feature map was created by merging the first and second feature maps, with a length of 392.

FS:

INCA: The range of iterations was 14 to 98, and the loss value generator was selected as kNN with tenfold CV. The attributes of this classifier are as follows:

- k: 1
- Distance: L1-norm
- Weight: equal

By applying the INCA feature selector, three selected feature vectors were created with lengths of 21, 25, and 80, respectively.

XAI:

DLob: DLob symbols described in Sect. "The presented SOXFE" were used to create DLob strings. Three DLob strings were created in this research based on the indices of the selected feature vectors. Each selected feature vector contains two DLob symbols. The lengths of the generated DLob strings are 42, 50, and 160, respectively. We used the transition table from the DLob symbols to build cortical connectome diagrams. Additionally, histograms and information entropies of the DLob symbols were determined.

Classification:

tkNN: Using this ensemble classifier, three classifier-based outcomes were constructed. The parameters for this ensemble classifier are as follows:

- Distance metrics: City block, Euclidean, Cosine, Spearman
- Weight: Equal, inverse, squared inverse
- k: from 1 to 10
- Number of constructed parameter-based outcomes: 120 (= 4 × 3 × 10)
- Number of constructed voted outcomes using IMV: 118
- IMV parameters:
- Range: 3 to 120
- Sorting factor: accuracy (descending)
- Voting function: mode
- Total number of outcomes: 238 (= 120 + 118)
- Selection of the best outcome: Based on maximum classification accuracy.

Information Fusion:

Mode-based majority voting: The mode operator was applied to the three tkNN-based outcomes, creating a voted outcome.

### Classification results

The proposed DMPat-based SOXFE approach generates both classification and explainable results. The first output of this SOXFE is the classification. Metrics such as classification accuracy, sensitivity, specificity, and geometric mean were used to evaluate the classification results. These performance metrics have been defined below, and to compute these classification metrics, the numbers of true positives (TP), false positives (FP), true negatives (TN), and false negatives (FN) have been used. The details of the performance metrics used in this research are given below (Chicco and Jurman 2020; Warrens 2008).

**Accuracy:** Accuracy illustrates how many predictions were correct out of all predictions. It defines how well the approach works overall. The formula for the classification accuracy is given below.

35 $$Accuracy=\frac{TP+TN}{TP+TN+FP+FN}$$

**Sensitivity/Recall:** Sensitivity showcases how many real positive cases the approach found. A high sensitivity means the approach catches most of the positive cases.

36 $$Sensitivity/Recall=\frac{TP}{TP+FN}$$

**Specificity:** Specificity demonstrates how many real negative cases the approach found. A high specificity means the approach avoids false alarms.

37 $$Specificity=\frac{TN}{TN+FP}$$

**Geometric Mean:** Geometric mean combines sensitivity and specificity. It gives a balanced score for both positive and negative classes. Geometric mean is helpful when the data is unbalanced.

38 $$Geometric Mean=\sqrt{\frac{TP}{TP+FN}\times \frac{TN}{TN+FP}}$$

By utilizing the four given classification performance metrics, the recommended DMPat-centric SOXFE has been evaluated.

This DMPat-based SOXFE approach generates four outcomes and selects the best one from the generated outcomes. Additionally, tenfold and LORO CV methods were utilized, and the computed classification performances are presented in Table 2.

**Table 2:** Results (%) of the presented DMPat-based SOXFE approach

Validation	Output	Acc	Sen	Spe	GM
LORO CV	1	97.12	96.85	97.29	97.07
	2	94.64	96.50	93.44	94.96
	3	98.08	98.60	97.74	98.17
	**4 (Voted)**	**98.49**	**98.60**	**98.42**	**98.51**
Tenfold CV	1	99.59	98.95	100	99.47
	2	99.31	99.30	99.32	99.31
	3	99.86	99.65	100	99.83
	**4 (Voted)**	**100**	**100**	**100**	**100**

**Acc.: Classification accuracy, Sen..: Sensitivity, Spe.: Specificity, GM: Geometric mean. The results of the final outcome have been highlighted using bold font face

**Fig. 4:**
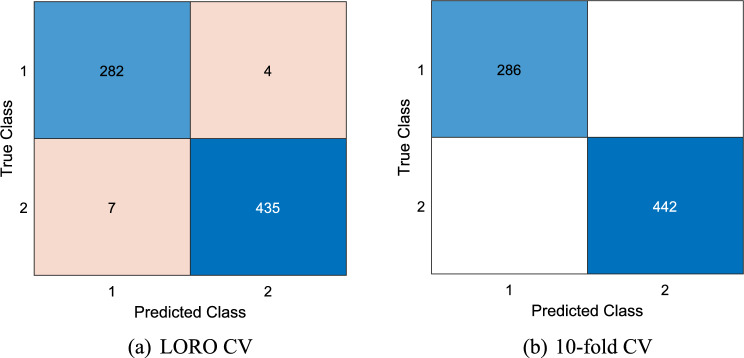
Confusion matrices of the final outcomes. Herein, 1: violence, 2: control

To give results comprehensively, confusion matrices of the final outcomes have been depicted in Fig. 4.

### Interpretable results

In the second step, the recommended DMPat-based SOXFE approach generates explainable results using DLob, and the indices of the selected feature vectors are utilized. Therefore, the generated three DLob strings are demonstrated in Table 3.

**Table 3:** The extracted explainable results on the used dataset, deploying DMPat-based SOXFE

Selected feature vector	DLob string	Cortical connectome diagram	Entropy
1	TR PL OR OR PL PL FR OL FL FL FR OL FR FR FR OR FR PL OR OL FR OL FR PL FR FR FR FR FR FR FR FR FR FR TR OR FR FL FR PR PR OL		1.4341
2	PL TL TR TL TL TL PR TL FR TL OR TL OL TL FR TL TR FL PL FL PL FL OL FL PR FL TL FL PR FL OL FL FR FL FR TL PR OR FR TR TL FL FR FL PR OL FR FL TR FL		1.9791
3	FL FL PL PL TL TL OR TL PR TL TR TL FR OL PL FL TR PL FR TL OR OR FR OL OL TL PL FL FR OR PR PR TR OR FR OL TR TR OR OL FR FL FR FR OL FL FR OR PR OR FR TR FR PL FR FR FR TL FR FL FR PL FR FR PL TL FR PR FR FR FL FL FL FL OR PL FR FL FR FR FR FR FR FR PR PL FL FL TR FL FR TR FR PL FL FL PR OL FR PL TR PR FR PR FR PR FR FL TR OL PR FL FR FR TL FL FR OR TL FL OL OL OR FL FR TR PL FL TR FL FR FL FR OL FR PR FR FR FR OR TL FL OL PL FR FL PR FL FR FL FR TL FR FR PR FL OR FL TR TL		1.9534

**Fig. 5:**
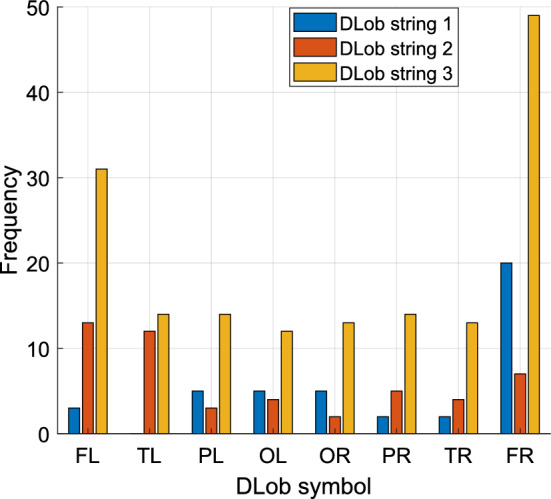
Frequencies of the used DLob symbols according to the DLob string

Table 3 showcases the generated DLob strings, cortical connectome diagrams, and information entropies of these generated strings. A DLob string was created for each selected feature vector. By computing the transitions of these three DLob strings, three cortical connectome diagrams were also created and showcased in Table 3. Moreover, the histogram of the used symbols is demonstrated in Fig. 5.

Figure 5 showcases that the dominantly active brain lobe is the frontal lobe, as the most frequently used DLob symbols are FL and FR.

## Discussion

In this research, we presented a new DMPat-based SOXFE approach, which is self-organized and explainable. This approach is considered self-organized because:

- INCA automatically selects the best feature vector,
- tkNN automatically selects the best classification outcomes,
- In the information fusion phase, the best final outcome is automatically selected.

Our essential motivation in this research is to extract information related to violence. Therefore, we collected a new EEG VD dataset from 14 participants. By collecting this dataset, we have integrated neuroscience with information security. Using this dataset, individuals who have experienced violence can be detected, and more approaches and datasets can be developed and collected to achieve this objective.

**Fig. 6:**
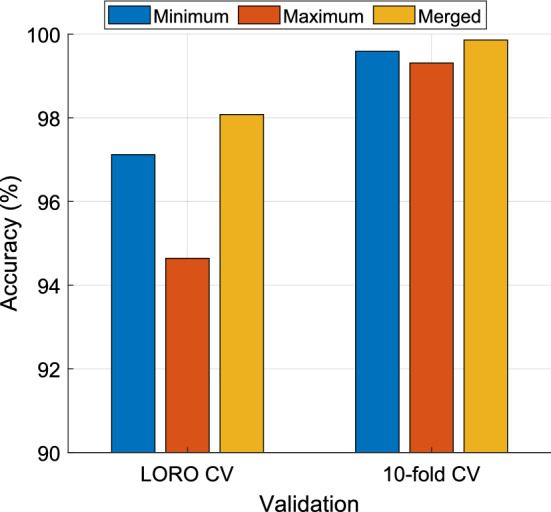
Classification accuracies of the feature vectors. Herein, the first feature vector was generated by deploying the minimum distance, the second feature vector was created using the maximum distance, and the third feature vector was the merged version of the first and second feature vectors

The DMPat feature extractor was proposed in our approach to attain high classification performance. The DMPat feature extractor identifies the maximum and minimum distances between channels, and using this information, it extracts valuable features. Furthermore, three feature vectors were extracted by deploying the recommended DMPat feature extractor. The classification performance of these feature vectors was investigated using tkNN-based classification accuracies, and the comparative results for the feature vectors are depicted in Fig. 6.

Figure 6 demonstrates that the best results are obtained from the third feature vector, which was created using the merging strategy. Furthermore, the minimum distance-based feature vector performs better than the maximum distance-based feature vector.

We aimed to achieve high classification performance by deploying INCA, tkNN, and information fusion. The recommended approach attained 98.49% and 100% classification accuracies using LORO CV and tenfold CV, respectively.

To present explainable features, we integrated the presented DMPat-based SOXFE approach to obtain explainable results. In Sect. "Performance evaluation", we presented the explainable results. The discussions for the generated DLob strings are as follows:

**DLob String 1:** The Occipital Lobe is highly engaged, reflecting the intense visual processing required when viewing violent videos. This is expected as the occipital cortex is responsible for visual perception. Overstimulation in this region could lead to a spillover effect, influencing other brain regions such as the parietal lobe (PL), which integrates sensory information and spatial reasoning. The high frequency of FR activations suggests that motor planning and cognitive processing are heavily involved. In violent situations, the brain's frontal regions are activated to prepare for potential movement (e.g., jumping or defensive actions) and to process complex information about the stimuli. Temporal Lobe involvement indicates emotional processing and memory retrieval. The temporal cortex plays a role in understanding the emotional content of videos, contributing to how the brain processes violent content.

**DLob String 2:** There is an increased involvement of both frontal and temporal lobes, suggesting significant cognitive and emotional processing while watching violent videos. The temporal lobe’s activation further indicates that the content may evoke strong emotional reactions and perhaps stimulate memory recall of similar experiences. The involvement of the occipital lobe remains consistent, reinforcing the importance of visual input processing. Meanwhile, the parietal lobe’s involvement reflects the brain’s integration of sensory and motor responses during the observation of violent stimuli.

**DLob String 3:** The frontal and parietal lobes remain key players, indicating sustained cognitive processing and motor planning, alongside the integration of sensory information. The brain appears to prepare itself for action, as would be expected during high-stress situations such as violence. Emotional responses continue to be processed, as the temporal lobe shows frequent activation. This suggests that the emotional impact of violent videos is substantial, influencing both immediate reactions and longer-term memory associations.

The findings, advantages, limitations and future works of this research are discussed below.

Findings:

- Three feature vectors were created by calculating maximum and minimum distances between channels in the EEG signals. Per the results, minimum distance-based features are more effective than maximum distance-based features. Also, the best feature vector is the merged feature vector.
- The INCA feature selector reduced the feature set to vectors of 21, 25, and 80 features.
- DLob symbolic language was used to generate cortical connectome diagrams, making the brain’s response to violence interpretable.
- Dominant in processing complex cognitive functions (FL and FR), including decision-making and motor planning. Their frequent activation during violent video viewing suggests heightened readiness for action, such as defensive movements or quick reactions.
- OL and OR symbols depicted high activation, indicating that the brain’s visual systems are heavily engaged when processing violent stimuli.
- Increased activation in PL and PR symbols reflects how the brain coordinates sensory inputs, particularly when combining visual information with potential motor actions during violence.
- TL and TR symbols are crucial role in emotional processing and memory retrieval. The emotional content of violent videos likely triggers responses in the temporal cortex, influencing both immediate emotional reactions and longer-term memory associations with violent experiences.
- Entropy values calculated from the DLob strings showcased moderate predictability (1.4341 to 1.9791).
- This approach uses three self-organized methods: INCA, tkNN and information fusion.
- The mode-based majority voting system and greedy algorithm selected the best classification result for both validation techniques.
- By extracting cortical connectome diagrams the cortical pathways of violence have been demonstrated.

Advantages:

- tenfold CV and LORO CV have been used to demonstrate robust and reliable classification performances and the recommended DMPat-based SOXFE approach reached 100% classification accuracy with tenfold CV and 98.49% with LORO CV.
- The DMPat-based FEX function and the overall approach have linear time complexity, making it efficient for real-time processing.
- Integration with the DLob symbolic language allows for extracting explainable results, bridging neuroscience and machine learning.
- It is the pioneering brain-related VD work and it opens new avenues for creating VD approaches for children and people with disabilities. In this aspect, this project is a social project.

Limitations:

- The approach was tested on a dataset of only 14 participants, which may not fully represent diverse populations or varied types of violent stimuli.
- More diverse and bigger EEG signal datasets can be used to test the presented DMPat-based SOXFE approach.

Future works:

- To the best of our knowledge, no public EEG dataset available that supports a detailed multiclass emotion detection task like our dataset (the available EEG emotion datasets do not include a violence class). We plan to collect data from more people with different backgrounds. The experiments should also be designed to trigger different emotions. This type of dataset would help build better emotion detection models and may help to understand the processing of the brain during different emotions better.
- The approach could be tested with a wider variety of violent stimuli to understand how different types of violence affect brain activity.
- Additional EEG channels can be incorporated to capture more detailed brain responses and improve the accuracy of the approach.
- The presented DMPat-based SOXFE approach's performance on real-time VD could be investigated in practical applications such as security systems or psychological assessments.
- Hybrid models could be developed by combining DMPat with other FEX methods to further enhance classification accuracy.
- The integration of the approach with other modalities, such as fNIRS or facial recognition, could be explored to provide a more comprehensive analysis of emotional and cognitive responses to violence.
- The use of the DLob symbolic language could be extended to broader contexts like detecting aggression or other emotional states.
- How the approach performs across different age groups, particularly in children or individuals with mental health conditions, could be investigated.
- The potential for deploying this approach in clinical settings to identify individuals with a predisposition to violent behavior or to support rehabilitation programs could be researched.
- A user-friendly software interface could be developed for non-experts to apply the approach in various real-world environments, such as schools, workplaces, or homes.
- We are planning to create a dictionary of the DLob for different brain activities.

## Conclusions

The DMPat-based SOXFE approach has demonstrated high classification accuracy in detecting violence through EEG signals. The approach achieved 100% accuracy using tenfold CV and 98.49% accuracy with LORO CV and these results highlight the robustness and reliability of the recommended DMPat-based SOXFE approach. By utilizing the DMPat FEX method, which calculates minimum and maximum distances between EEG channels, and integrating the INCA feature selector, the approach effectively reduced the feature space while maintaining strong performance. The merged feature vector, combining both minimum and maximum distance-based features, proved to be the most effective.

The DLob symbolic language was employed to generate cortical connectome diagrams, making the brain's response to violent stimuli interpretable. The frontal lobes (FL and FR) were shown to play a key role in decision-making and motor planning, while occipital lobes (OL and OR) were heavily engaged in processing visual information. The parietal lobes (PL and PR) coordinated sensory input, and the temporal lobes (TL and TR) contributed to emotional processing and memory recall. This integration of machine learning and neuroscience through XAI bridges the gap between these fields, offering a deeper understanding of brain activity during violent stimuli and opening avenues for future research and practical applications.

## Data Availability

No datasets were generated or analysed during the current study.
